# TG and *G&D*: some thoughts and reminiscences

**DOI:** 10.1101/gad.350473.123

**Published:** 2023-01-01

**Authors:** Michael B. Mathews

**Affiliations:** Department of Medicine, Rutgers New Jersey Medical School, Newark, New Jersey 07103, USA

Terri and I met at Cold Spring Harbor Laboratory nearly half a century ago as junior scientists in the James lab. Both of us were studying adenovirus. Our work was complementary and we wrote three papers together, dealing with viral genes that encoded regulatory proteins and RNAs. The last of these papers, coauthored with Elizabeth Moran, Rich Roberts, and Brad Zerler, was on gene expression and cellular transformation induced by products of the virus's E1A gene. It was published in the *Journal of Virology* in 1986. Three eventful years later, Terri was US Editor of *Genes & Development*, a role she has fulfilled in exemplary style for over a third of a century. Let me recount the events that led to her stewardship of *G&D*.

Terri took over the editorship from me in 1989, relieving me of the position I had held for about 2 years following the tragic and singularly untimely death of Steve Prentis. Steve founded *G&D* as a “journal devoted to the molecular analysis of gene expression in eukaryotes, prokaryotes, and viruses.” It was a joint venture with the Genetics Society of Great Britain, and Graham Bulfield was European Editor. True to its principles, the first issue, which came out in March 1987, featured papers and commentaries on plants, animals, and bacteria, as well as viruses infecting all three, from laboratories in Europe and (mainly) the United States. This was a notable launch, and the journal has continued to publish top-drawer science and gain respect in the field ever since.

When I took on the position, it was in the immediate aftermath of Steve's tragic death in a road accident, made all the more poignant as it befell on the verge of the appearance of the journal's very first issue. Many of us at CSHL became aware of the disaster as we were gathering the next morning for an in-house symposium in Grace Auditorium. I was on the newborn journal's Editorial Board and naturally—perhaps naïvely—offered to do what I could in the wake of this calamity. At the time, I had no real idea what was entailed, but Judy Cuddihy was there to steady the ship. (Somewhat confusingly, her title was Editor, while Steve and Graham were Executive Editors; these things have changed since.) Judy had worked closely with Steve to bring the journal into being and had a clear sense of what it was intended to be as well as the skills to manage it and keep the monthly production on schedule.

Being Editor was an education for me, but to all appearances the journal thrived. This was not without a few hiccups, however. Selection of the cover art was sometimes a source of debate in the journal office upstairs in the Carnegie Library building. One cover with a plant theme drew more discussion than usual and has occasioned amused comments from Terri (though a not dissimilar illustration graces a CSHL Symposium volume that she coedited!). Needless to say, I was indebted to our Editorial Board members and outside referees for outstanding and knowledgeable guidance, but I was also conscious of the journal's announced scope and high aspirations. This led to a few confrontations. One Nobel laureate, whose paper was accepted, grumbled about my insistence on the distinction between the rate of synthesis of a macromolecule and its accumulated level (though he yielded to persuasion in the end). Another luminary complained that I'd had the audacity to reject his paper. The word came back to me from Jim Watson, possibly through Terri, that the journal—or was it me?—couldn't survive more than one incident a year of this type. I hasten to add that Jim took a keen interest in the journal and its progress but made no move to influence its running. It was left to sink or swim.

The journal swam, of course, but I wanted to get back to the lab full time. John Inglis arrived at CSHL as director of the Press—the position Steve had held while spearheading *G&D*—but not as Editor of the journal. Fortunately, Terri came to the rescue and offered to take on the job of editing *G&D*. She was eager to do it and no less qualified than I was when I took it on (nor discernibly more so, either), but still, would she take to it, we wondered? The journal was a going concern, after all. Shouldn't we do something to gain reassurance that it would be in good hands? To Terri's faintly disguised amusement, Judy and I devised a trial run of sorts. Terri was handed a few manuscripts and their reviews to evaluate and make recommendations on: to accept for publication, return for revisions either major or minor, or decline. She did so; we pontificated and, with some relief on my part, we approved. I'll never forget Terri's quizzical response, transmitted nonverbally but unmistakably. To this day, it reminds me of John Lennon's tongue-in-cheek closing remark on the Apple building rooftop: “I'd like to say thank you on behalf of the group and ourselves, and I hope we've passed the audition.” Of course she took to it, and the journal and community it serves are the better for it.

For Terri, this was the beginning of a remarkable tenure, during which she deftly stewarded the journal, enhanced its reputation, published great science, and saw it flourish. We met over the years, often at conferences where Terri was a focus of bonhomie as well as serious scientific business. Congratulations on a marathon stint as Editor, Terri! May the future be equally successful and fulfilling.

